# Surgical application of the keystone island flap for closure of thoracolumbar myelomeningocele defects - A case report

**DOI:** 10.1016/j.amsu.2020.08.012

**Published:** 2020-08-13

**Authors:** Mohamed Amir Mrad, Ahmad Alharbi, Nehal Mahabbat, Atif Rafique, Fuad Hashem

**Affiliations:** Plastic and Reconstructive Surgery Section, Surgery Department, King Faisal Specialist Hospital and Research Centre, Saudi Arabia

**Keywords:** Neural tube, Myelo-meningocele, Fasciocutaneous, Keystone design perforator island flap (KDPIF)

## Abstract

**Introduction:**

Myelomeningocele (MMC) is the most common neural tube defect that can occur due to neural tube's failure to fuse properly during embryonic life. To prevent this, keystone island flap can be used for closure of large MMCs.

**Presentation of case:**

A new-born girl born as a product of 36 weeks of gestation had a weight of 3.020 kg and had multiple congenital anomalies including hydrocephalus, thoracolumbar myelomeningocele at the level of (T10-L4) and an atrial septal defect. Preoperative evaluation showed a head circumference of 42 cm (n: mean 34.4 ± 2SD), no lower limbs movements and a thoracolumbar soft tissue defect around 4 × 8 cm with exposed neuronal tissue and prominent thoracic kyphosis, and no obvious urogenital or limbs anomalies. The large thoracolumbar myelomeningocele was treated at KFSHRC with a Keystone Design Perforator Island Flap (KDPIF) to reconstruct the soft tissue defect following the neurosurgical reconstruction.

**Discussion:**

The keystone flaps were deemed as viable as all wounds were healed without any complications, such as flap necrosis, dehiscence, leakage of cerebrospinal fluid, or infection. The technique described in the case report offers a simple and effective method of wound closure in situations that would, otherwise, have required complex flap closure.

**Conclusion:**

This flap can be an effective method for reconstruction of large thoracolumbar MMC defects that might improve outcome and minimize complications. It also ensures good watertight closure with minimal wound tension and breakdown.

## Highlights

• Myelomeningocele is marked by failed fusion of neural tubes during embryonic life.
• Keystone Design Perforator Island Flap can be used to close large myelomeningocele.
• It is a simple and an effective method of wound closure without a complex flap usage.
• It is used to distribute wound tension over the whole defect, especially over the midline.
• It ensures good watertight closure with minimal wound tension and breakdown.

## 1 Introduction

Myelomeningocele (MMC) is one of the more occurring and catastrophic neural tube defects with annual incidence rates of 0.5–1.0/1000 pregnancies in the United States and about 4 per 10,000 births worldwide [[1], [2], [3], [4]]. This defect is characterized by incomplete development of the embryonic neural plate, which does not close at some point along its length with subsequent herniation of both neural and meningeal elements through the opening in the spine [5]. About 90% of patients with this condition have other related problems, such as hydrocephalus and Arnold-Chiari malformation, which require a VP shunt placement [6,7]. Moreover, this kind of defect is usually complicated by long-term comorbidities such as bowel and bladder dysfunction, paralysis, and various degree of mental retardation which can reach up to 15% [8].

MMC remains to be one of the most noteworthy serious birth defects, despite the decline in incidence of open neural tube defects as credited to peri-conceptional folic acid supplementation among childbearing age women. To treat this, utmost priority is given to the prevention of central nervous system infection as well as protection of the exposed nerves and structures from additional trauma. As such, early closure is recommended and is ideally within 48 hours of birth to eliminate these risks [9]. Although most wounds are small enough to be closed mainly with simple soft tissue approximation, local flaps may be needed in about 25% of cases where the defects are deemed as large [9]. Transposition of local tissues with similar qualities is needed to achieve the closure of the skin defect following repair in an ideal manner. Several techniques of closure and reconstruction of this kind of defect have been the focus of discussion of much of the literature.

In this case report, we describe a local flap – the Keystone Design Perforator Island Flap (KDPIF) – to reconstruct soft tissue defects after repairing the thoracolumbar myelomeningocele defect. It is designed as a curvilinear shaped trapezoidal flap, which is borrowed from the Roman architectural terminology. It is made up of skin, subcutaneous fat and deep fascia together with its randomly located musculo/fasciocutaneous perforators [10]. Its lateral edges are extended with acute angles and by V–Y advancement of each end of the flap with undermining of the surrounding tissues to fill the defect. The flap is designed to distribute wound tension among the defect, particularly over the midline area of maximum tension which overlies the primary defect. Wound complications, including cerebrospinal fluid (CSF) leak, infection and contraction of soft tissue has been reported in many studies, the reported CSF leak was 1–3% after myelomeningocele repair [11,12].

## 2 Case presentation

In this case report, a female neonate, with a weight of 3.020 kg, was born as a product of 36 weeks of gestation through a spontaneous vaginal delivery. She had multiple congenital anomalies including hydrocephalus, thoracolumbar myelomeningocele at level of (T10-L4) and an atrial septal defect. The neonate Apgar score was recorded to be 8 (1 min) and 9 (5 min) post-delivery. The patient was managed by a multi-disciplinary team to reach an optimal outcome. Her immediate postnatal period was in a NICU setting on mechanical ventilation with respiratory distress and multiple electrolytes disturbances.

The evaluation showed a head circumference of 42 cm (n: mean 34.4 ± 2SD), no lower limbs movements and a thoracolumbar soft tissue defect around 4 × 8 cm with exposed neuronal tissue and prominent thoracic kyphosis. No obvious urogenital or limbs anomalies were observed. Pre-operatively, the defect was covered with non-adherent moist dressing and the patient was covered with broad spectrum antibiotics.

• **Surgical procedure - description of the operative technique**:

Double opposing keystone island perforator flaps were deemed the reconstructive option of choice. The patient was taken to the operative theatre on day 4 postnatally, placed lying on the operating table. Prepping of the whole back, including the neck and the gluteal regions, was carried out.

Flaps were designed on the edges of the defect, with each flap's width equal to the width of the defect. From the superior and inferior tips of the defect, 90-degree limbs extending bilaterally will be connected to the trapezoidal shaped flaps (as a type 3 keystone flap-originally described by Behan). Throughout the vertical dimension of the flap, each flap width was maintained to be equal to the defect width. The neurosurgery team started initially by dissecting the neural sac (placode) off the surrounding tissue and performing a complete watertight primary closure of the pseudo-dural sac.

For the reconstructive part, the fascia was incised over the spinous processes on the medial edge of the flap. This was succeeded by incising the lateral edge of the flap and the dissection continued to the subcutaneous tissues and lumbar fascia down to but not through the muscles. Both lateral and medial edges of the flaps were undermined minimally under vision at the sub-fascial plane to facilitate the flap mobility without exceeding more than 5 mm undermining and with the preservation of the thoracolumbar perforators on the medial edge a drain was inserted in the dead space.

Primary closure was performed in two layers, with moderate amount of tension on both medial and lateral edges of the flap. Likewise, the secondary defects on both sides were also closed primarily with moderate amount of tension. No dressing was applied and daily ointment application on flap edges was done.

Total operative time of the plastic surgery team was one hour. After the procedure, the patient was admitted to the neonatal intensive care unit. A week post-operatively, a VP shunt was inserted, and the patient was extubated after 2 weeks without any complications, and was shifted to the ward after 3 weeks.

**Follow-up:** Removal of the drain was performed 1-week post-op. The patient was kept on prone and lateral positions strictly for 3 weeks, after which she was allowed to be positioned on her back. Follow-up period was conducted for a total period of 4 months.

No complications, including seroma, hematoma, CSF leak, wound infection, wound dehiscence, or wound edge necrosis, was observed during the follow-up period. The patient did not require any further surgical intervention regarding her defect closure and was discharged from the hospital after complete wound healing.

This case report was reported in line with the SCARE 2018 Checklist [13].

## 3 Discussion

Neural tube defects (NTDs) are one of the most common congenital anomalies of the central nervous system, which can occur in about 300,000 births each year [14]. Spina bifida can range from mild to severe, depending on the type of defect, size, location, and complications. Myelomeningocele (MMC) is deemed as the most severe and complicated form that is compatible with life [15,16].

Several closure techniques have been utilized for defect reconstruction. However, a tension-free skin closure in the midline remains to be one of the major considerations and a challenge in large MMCs. These techniques include primary closure, wide undermining with skin advancement, split skin graft, local flaps or lumbosacral fasciocutaneous flaps, transposed muscle flaps using latissimus dorsi, gluteal, trapezius or paraspinous muscles with split skin graft, musculocutaneous flaps with split skin grafting of secondary defect and perforator flaps namely superior gluteal artery perforators, and dorsal intercostal artery perforator flaps [11].

Large MMC defects closure has always been very difficult and is usuaully coupled with a high rate of complications. Keystone Design Perforator Island Flap (KDPIF) is an emerging reconstructive technique, with immense popularity in the reconstruction of various body defects. It was introduced by Behan in 2003 and was named as such due to its resemblance with the stone of Roman archways. Four main types have been described and reported in the literatures: Type I (unilateral keystone flap with direct closure), Type IIA (as in type I with additional division of the deep lateral fascia), Type IIB (as in type I with closure of the secondary defect using a split thickness skin graft), Type III (double keystone flaps), and Type IV (rotational keystone flap).

When selecting a reconstructive option, flap reliability is deemed as an important consideration. In 2006, Rubino had studied the blood flow hemodynamic changes that occur after perforator flap islanding and had compared it to the resting dynamics of cutaneous blood flow. He found out that blood flow and perforator diameter increased after flap elevation with the use of Doppler ultrasound measurement of vessel diameters, velocity, and flow, he named this finding as an inverse phenomenon [17].

In addition to the physiological changes that happen after flap islanding as described by Rubino, KDPIF maintains multiple conjoined vascular supply, including direct and indirect perforators, as well as cutaneous perforators coming from the nerves travelling toward the flap. In terms of vascularity, these properties increase the reliability of KDPIF [18].

In this case report, a bilateral keystone flap was used in the repair a large thoracolumbar myelomeningocele present in a newborn girl. The flap was intended to distribute wound tension over the defect predominantly over the midline which overlies the pseudo-dural closure. Basically, it is elliptical in shape with its long axis adjacent to the long axis of the defect. The flap width is deemed equal to the defect width and the length is usually determined by the size of the elliptical excision.

In contrast to other perforator flaps, this flap is technically simple and efficient as localization of specific perforators using Doppler US is deemed unnecessary. Limited undermining is generally performed only as much as necessary in order to facilitate flap movement while random musculocutaneous perforators of the flap are preserved.

Complications, such as seroma, hematoma, wound infection, wound dehiscence, flap necrosis or cerebrospinal fluid (CSF) leak, were not observed post-operatively. In an aesthetic perspective, the results were deemed as pleasing and acceptable as the curvilinear design of the keystone flap fits well into the body contour. Moreover, skin closure after defect repair was achieved via transposition of local tissues of similar qualities in terms of its color and contour.

Related findings on KDPIF have been successfully demonstrated in a study of 300 patients as to which KDPIF was applied over the extremities and trunk. Proper wound healing rate of 99.6% was achieved, with one out of 300 developing partial necrosis of the flap [10]. In a similar study including eight patients diagnosed with head and neck cancers, only one case reported sustained wound dehiscence [19].

In contrast, variable surgical options of coverage have been described and evaluated in various literatures. Lien and colleagues had utilized multiple flap options for large MMC defect closure. According to their research, patients who underwent simple closure, unilateral or bilateral rhomboid flap, rotational advancement flap had showed many post-operative complications, such as skin flap separation (20%), skin flap dehiscence (4%), skin flap necrosis (2%) and skin or subcutaneous infection (7%) [11]. Although arguments on Keystone Island Perforator Flaps are claimed to close a defect under tension as characterized by high rates of wound breakdown and edge necrosis, the design serves as an advantage as the tension is well tolerated via redistribution from the widest area of the defect across the whole length of the flap [18]. Using porcine biomechanical flap studies, Larrabee concluded that flap survival is ensured despite tension if the flap length is short. As KSIPF is characterized by having multiple perforators, no undermining, and short in design as compared to pedicled flaps, such advantageous characteristics make the patients tolerate a considerable amount of tension [18,20].

We believe that the technique described in this article offers a simple and effective method for large wound defects, as to which complex flaps have been deemed necessary in the past. Furthermore, the keystone flap will allow improvement of the results and reduce rates of complications associated with other kinds of flaps.

## 4 Conclusion

KDPIF is a unique and a reliable reconstructive option that is effective for locoregional fasciocutaneous reconstruction of large thoracolumbar MMC defects. Although no wound dehiscence post-operatively was reported in this case report, a larger case series of keystone flaps is to be conducted to assess the true incidence of wound dehiscence by this technique.

## Consent for publication

Written informed consent was obtained from LAR/parents/next of kin for publication of this case report and any accompanying images. A copy of the written consent is available for review by the Editor-in-Chief of this journal on request.

## Provenance and peer review

Not commissioned, externally peer reviewed.

## Sources of funding

The authors have no financial interest to declare in relation to the content of this article.

## Ethical approval

Ethical consideration was approved by the ethical committee of the surgery department of King Faisal Specialist Hospital and Research Centre.

## Author contribution

1. Dr. Mohamed Amir Mrad, MD, MBA, FRCSC, FACS – study concept, surgery
2. Dr. Ahmad Alhrbi, MBBS –writing the paper
3. Dr. Nehal Mahabbat, MBBS – data collection, writing the paper
4. Dr. Atif Rafique, FCPS, FEBPRAS –writing and paper review
5. Dr. Fuad Hashem, MBBS, FRCSC - study concept, surgery

## Registration of research studies

1. Name of the registry: Research Registry
2. Unique Identifying number or registration ID: 553
3. Hyperlink to your specific registration (must be publicly accessible and will be checked): https://www.researchregistry.com/browse-the-registry#home/registrationdetails/5e9e756f8d4c67001979432c/

## Guarantor

Dr. Mohamed Amir Mrad, MD, MBA, FRCSC, FACS.

Dr. Fuad Hashem, MBBS, FRCSC.

## Declaration of competing interest

The authors report no conflicts of interest. The authors alone are responsible for the content and writing of the paper.
