# Supplementary material for: Surgical application of the keystone island flap for closure of thoracolumbar myelomeningocele defects - A case report
Source: Ann Med Surg (Lond). 2020 Aug 13;57:339–42. doi: 10.1016/j.amsu.2020.08.012 (PMC7452152; doi:10.1016/j.amsu.2020.08.012)
Supplement: Multimedia component 1 [file mmc1.docx]

**Supplementary material:**


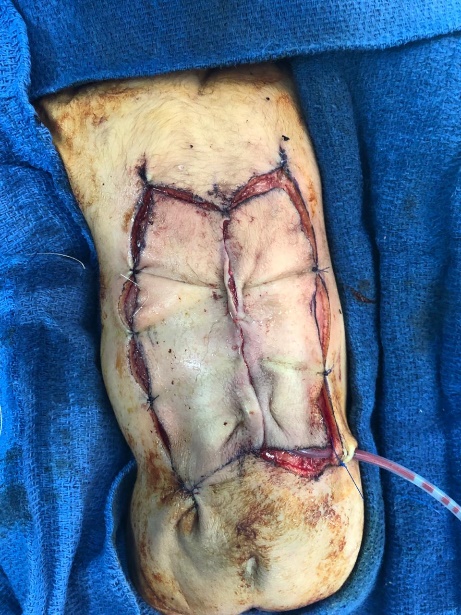

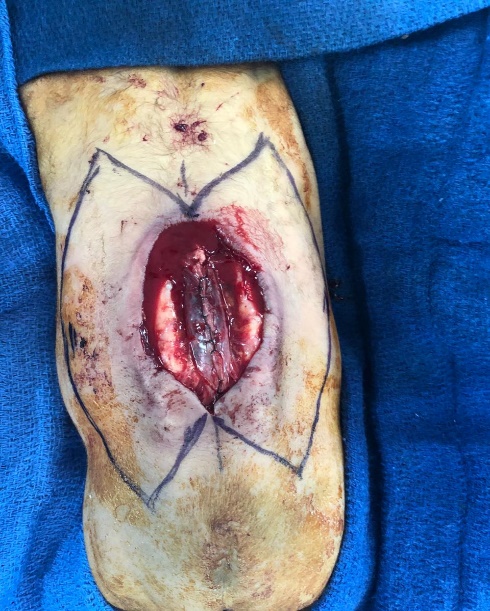

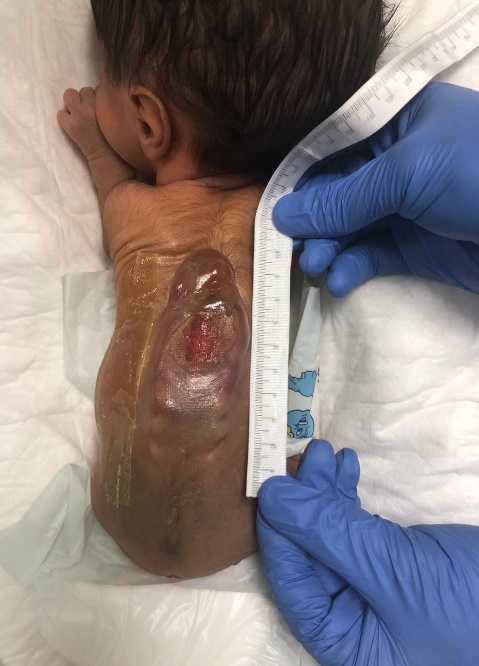


3

21

11

**Figure 1.** Pre-operative measurement view of the thoracolumbar myelomeningocele

**Figure 2.** The intra-operative view of the thoracolumbar myelomeningocele after closure of dura and curvilinear design of the Keystone Design Perforator Island Flap (KDPIF)

**Figure 3.** Intra-operative view after first tension sutures


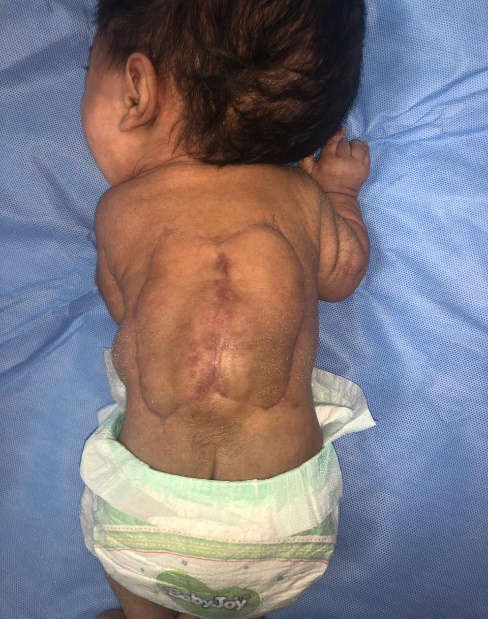

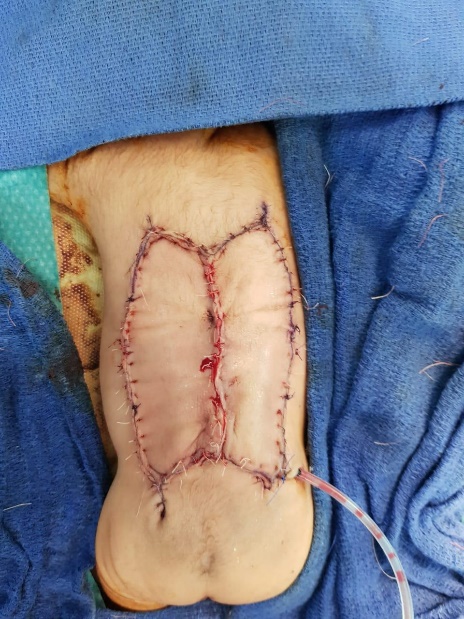

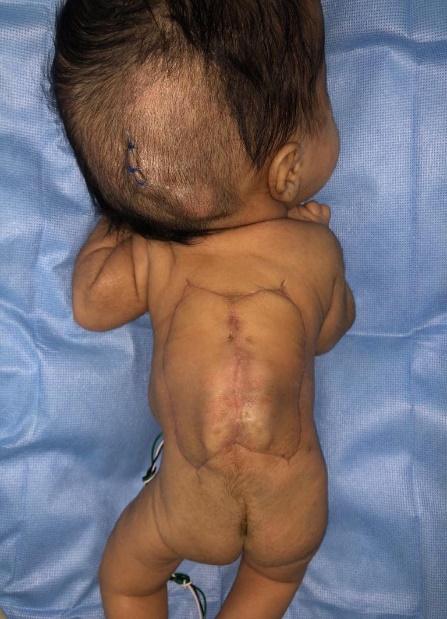


6

51

41

**Figure 4.** Results after full closure of the defect.

**Figure 5.** Results after 2 months post-operative.

**Figure 6.** Results after 4 months post-operative.
